# Supplementary material for: A Nanobody-Based Toolset to Monitor and Modify the Mitochondrial GTPase Miro1
Source: Front Mol Biosci. 2022 Mar 10;9:835302. doi: 10.3389/fmolb.2022.835302 (PMC8960383; doi:10.3389/fmolb.2022.835302)
Supplement: Supplementary file 1 [file DataSheet1.PDF]

A nanobody-based toolset to monitor and modify the mitochondrial GTPase Miro1

Funmilayo O. Fagbadebo[1], Philipp D. Kaiser[2], Katharina Zittlau[3], Natascha Bartlick[4], Teresa R. Wagner[1,2], Theresa Froehlich[1], Grace Jarjour[1], Stefan Nueske[5], Armin Scholz[5], Bjoern Traenkle[2], Boris Macek[3] and Ulrich Rothbauer[1,2#]

Affiliations/Addresses

[1] Pharmaceutical Biotechnology, Eberhard Karls University Tübingen, Germany

[2] NMI Natural and Medical Sciences Institute at the University of Tübingen, Reutlingen, Germany

[3] Quantitative Proteomics, Department of Biology, Interfaculty Institute of Cell Biology, Eberhard Karls University Tübingen, Germany

[4] Interfaculty Institute of Biochemistry, Eberhard Karls University Tübingen, Germany

[5] Livestock Center of the Faculty of Veterinary Medicine, Ludwig Maximilians University Munich, Oberschleissheim, Germany

[#] corresponding author

Prof. Dr. Ulrich Rothbauer, Natural and Medical Sciences Institute at the University of Tübingen

Markwiesenstr. 55, 72770 Reutlingen, Germany.

E-mail: ulrich.rothbauer@uni-tuebingen.de

Phone: +49 7121 51530-415

Fax: +49 7121 51530-816

Orcid ID: 0000-0001-5923-8986

Supplementary information

Supplementary Data

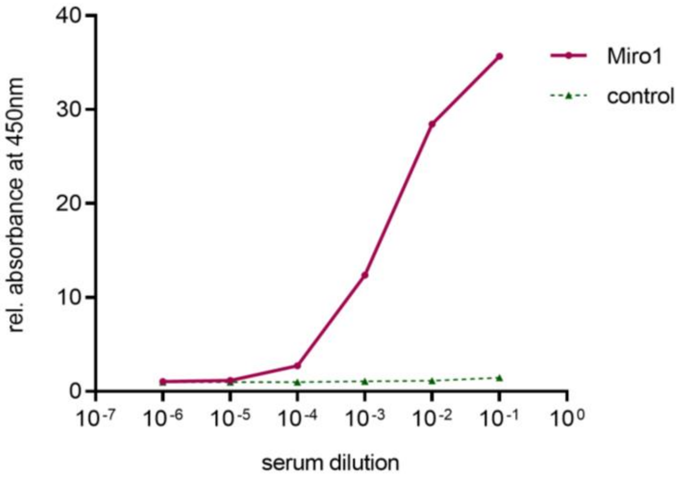

Supplementary Figure 1. Analysis of seroconversion upon vaccination with Miro1. To monitor an immune response upon vaccination, a serum sample was taken from the immunized Alpaca (*Vicugna pacos*) on day 63 after starting immunization. Formation of Miro1 specific antibodies by the animal was measured in a serum ELISA at indicated dilutions in multiwall plates either coated with hMiro1 or bovine serum albumin (BSA) as negative control. Bound antibodies were detected using an anti-heavy chain antibody and a secondary antibody labelled with horse radish peroxidase. Obtained ELISA signals for hMiro1 were normalized to signals obtained for the negative control.

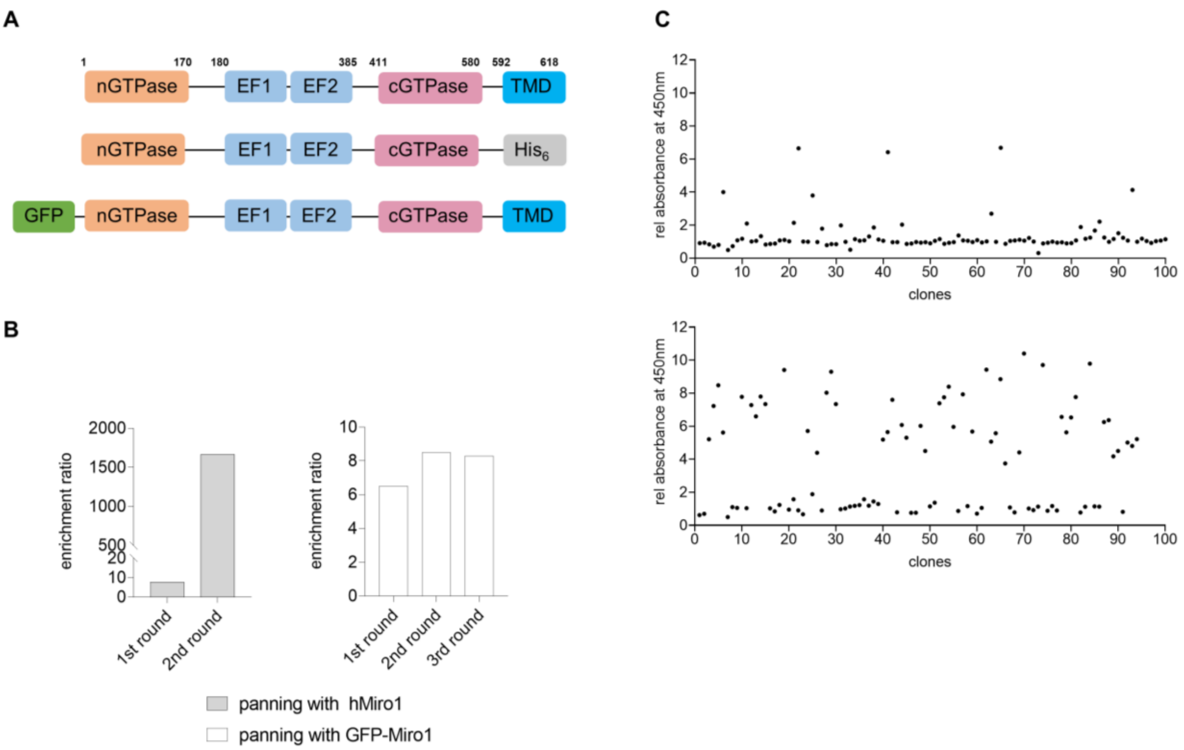

Supplementary Figure 2. Enrichment and selection of Miro1 nanobodies (Nbs) by phage display and phage ELISA.

(**A**) Illustration of wildtype Miro1 and recombinant Miro1 constructs used for phage display. (**B**) Bar charts showing the enrichment of Miro1-Nb phages after two iterative panning rounds against bacterial expressed hMiro1 (left panel, grey bars) and three iterative panning rounds against GFP-Miro1 (right panel, white bars). (**C**) Phage ELISA profile of 100 eluted phage clones tested for binding to hMiro1 (top panel) or GFP-Miro1 (lower panel). Signal intensities were normalized to a signal obtained for BSA used as negative control.

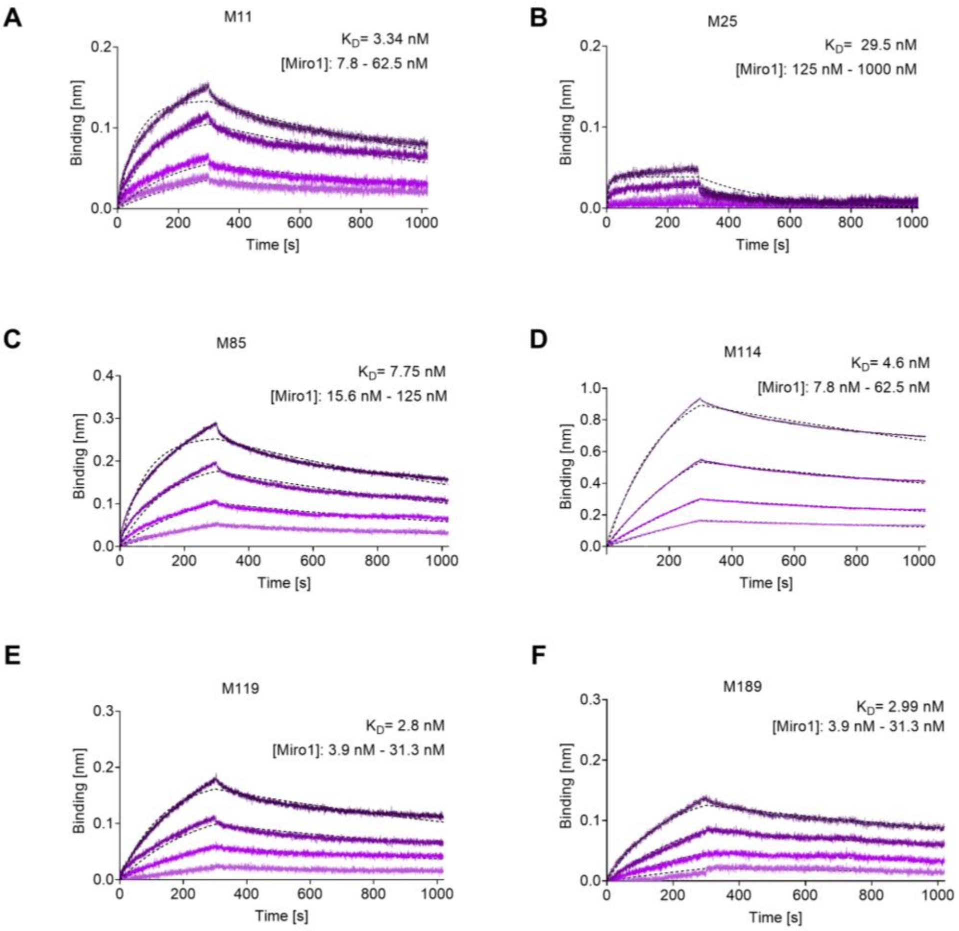

Supplementary Figure 3. Affinities of identified Miro1-Nbs.

Affinities of Miro1-Nbs were analysed by biolayer interferometry based affinity measurements. Miro1-Nbs were biotinylated and immobilized on streptavidin sensors. Kinetic measurements were performed by using four concentrations of hMiro1. The sensograms of hMiro1 on M11 (**A**), M25 (**B**), M85 (**C**), M114 (**D**), M119 (**E**) and M189 (**F**) at indicated concentrations (illustrated with increasingly darker shades from low to high concentration) are shown and global 1:1 fits are illustrated as dashed lines. A summary of the affinities (K_D), association constants (K_{ON}) and dissociation constants (K_{OFF}) determined for all seven Miro1-Nbs are shown in **Figure 1C, Table 1**.

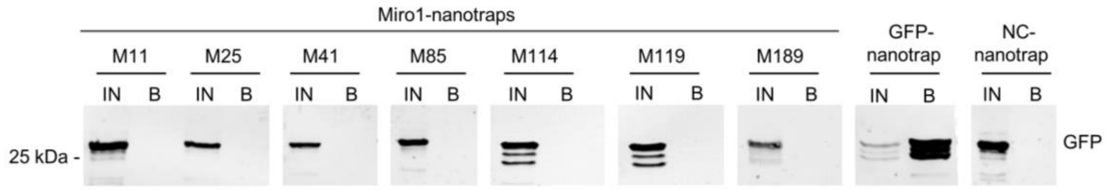

Supplementary Figure 4. Immobilized Miro1-Nbs (nanotraps) do not bind GFP.

Soluble protein fraction of HEK293 cells transiently expressing GFP as control antigen, was adjusted to 2 mg/mL and incubated with equal amounts of nanotraps. Input (IN, 1% of total) and bound (20% of total) fractions were subjected to SDS-PAGE followed by immunoblot analysis using antibodies specific for GFP. As positive control GFP-nanotrap and as negative a non-specific (NC) nanotrap were used.

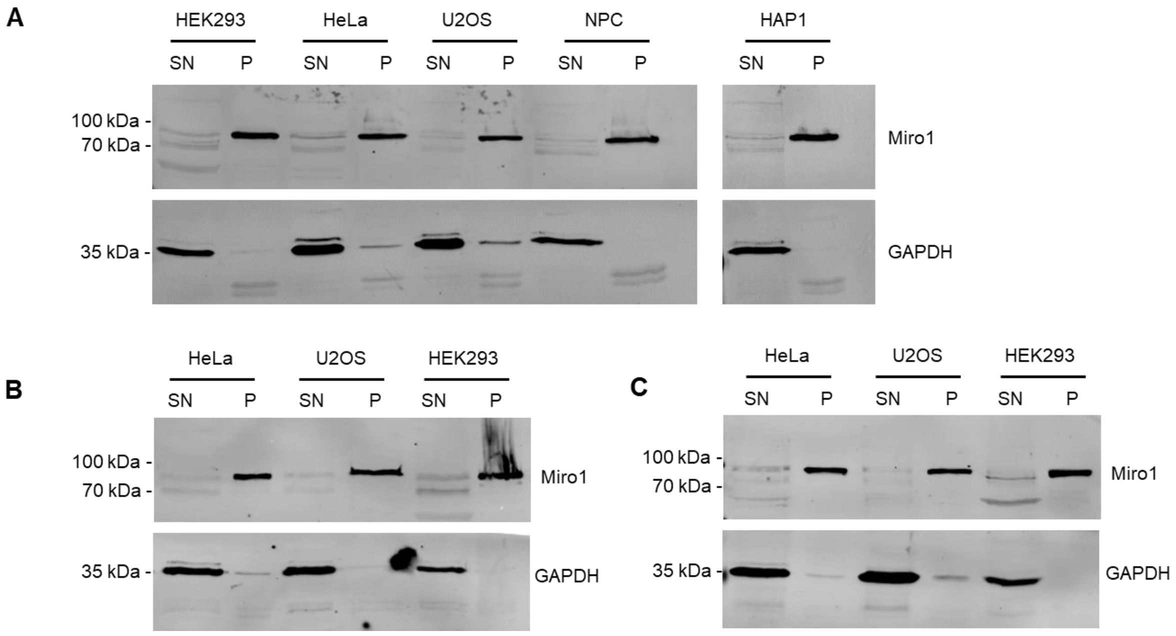

Supplementary Figure 5. Endogenous Miro1 retains in the insoluble protein fraction in different cell lines.

Representative western blot analysis of 20 µg of soluble (SN) and insoluble/pellet (P) fractions of indicated cells after addition of 0.5% NP-40 (**A**), or 1% Triton X-100 in the lysis buffer (**B**), or upon lysis in denaturing radioimmunoprecipitation assay (RIPA) buffer (**C**). Upper part of the blots were detected with anti-Miro1 antibody. Detection of GAPDH with an anti-GAPDH antibody was used as lysis and loading control.

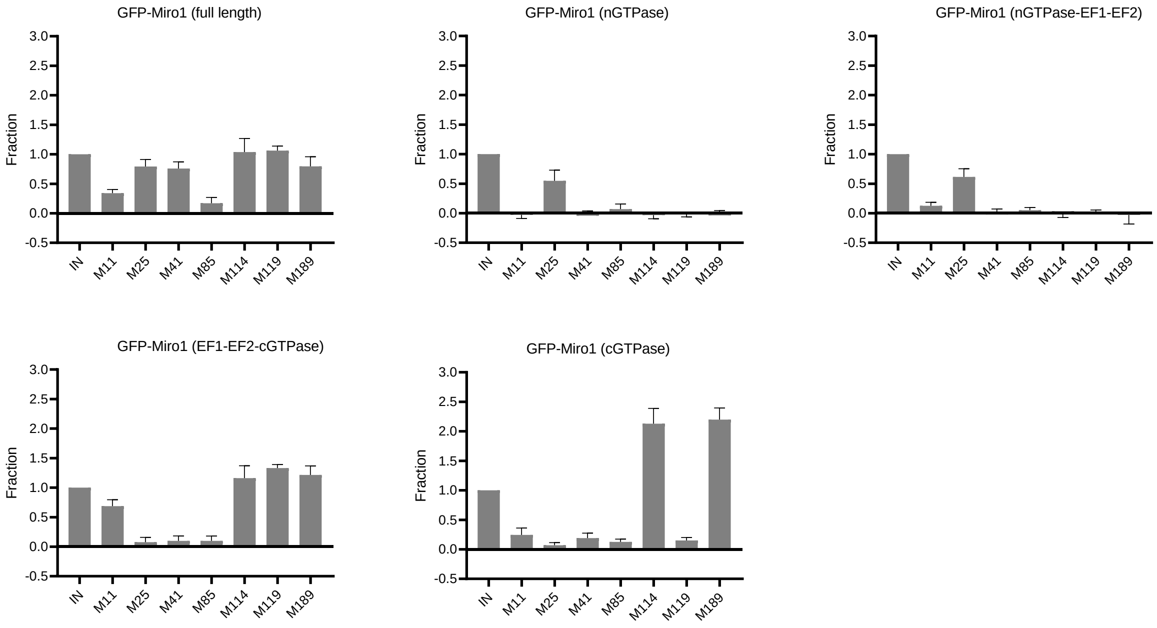

Supplementary Figure 6. Quantification of enriched GFP-Miro1 and Miro1 domains in bound fractions of Miro1 nanotraps

Densitometric evaluation of immunoblots shown in **Figure 3B.** The protein levels detectable in the bound fractions using an anti-GFP antibody were normalized to the input fraction. Shown are the mean signals from three independent experiments ± S.D.

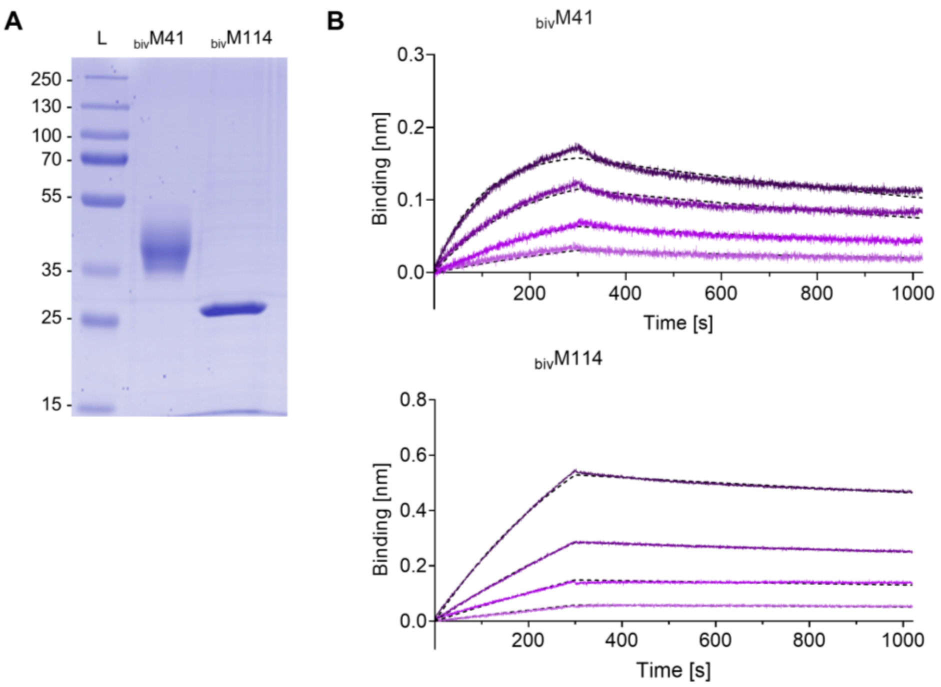

Table 1

	K_D (nM)	k_{ON} (10^5 M^{-1} s^{-1})	k_{OFF} (10^{-4} s^{-1})	R^2
**$_{biv}$M41**	1.86	3.19 ± 0.02	5.95 ± 0.02	0.99
**$_{biv}$M114**	2.35	0.78 ± 0.00	1.83 ± 0.01	0.99

Supplementary Figure 7. Recombinant expression, purification and characterization of bivalent M41- and M114-Nbs.

(**A**) Coomassie stained SDS-PAGE of 2 µg $_{biv}$M41- and $_{biv}$M114-Nbs purified from ExpiCHO™ cells is shown. Differences in running behaviour is due to N-glycosylation of bivM41 upon expression in mammalian (ExpiCHO) cells. (**B**) Affinities of bivalent Miro1-Nbs were analysed by biolayer interferometry (BLI) based affinity measurements. Bivalent Miro1-Nbs were biotinylated and immobilized on streptavidin sensors. Kinetic measurements were performed by using four concentrations of hMiro1 ranging from 3.9 nM – 31.3 nM (illustrated with increasing concentrations in darker shades). The sensograms of purified Miro1 on $_{biv}$M41-Nb (top) and $_{biv}$M114-Nb (bottom) are shown and global 1:1 fits are illustrated as dashed lines. The table summarizes affinities (K_D), association (k_{ON}) and dissociation constants (k_{OFF}), and coefficient of determination (R^2) determined for both bivalent Nbs.

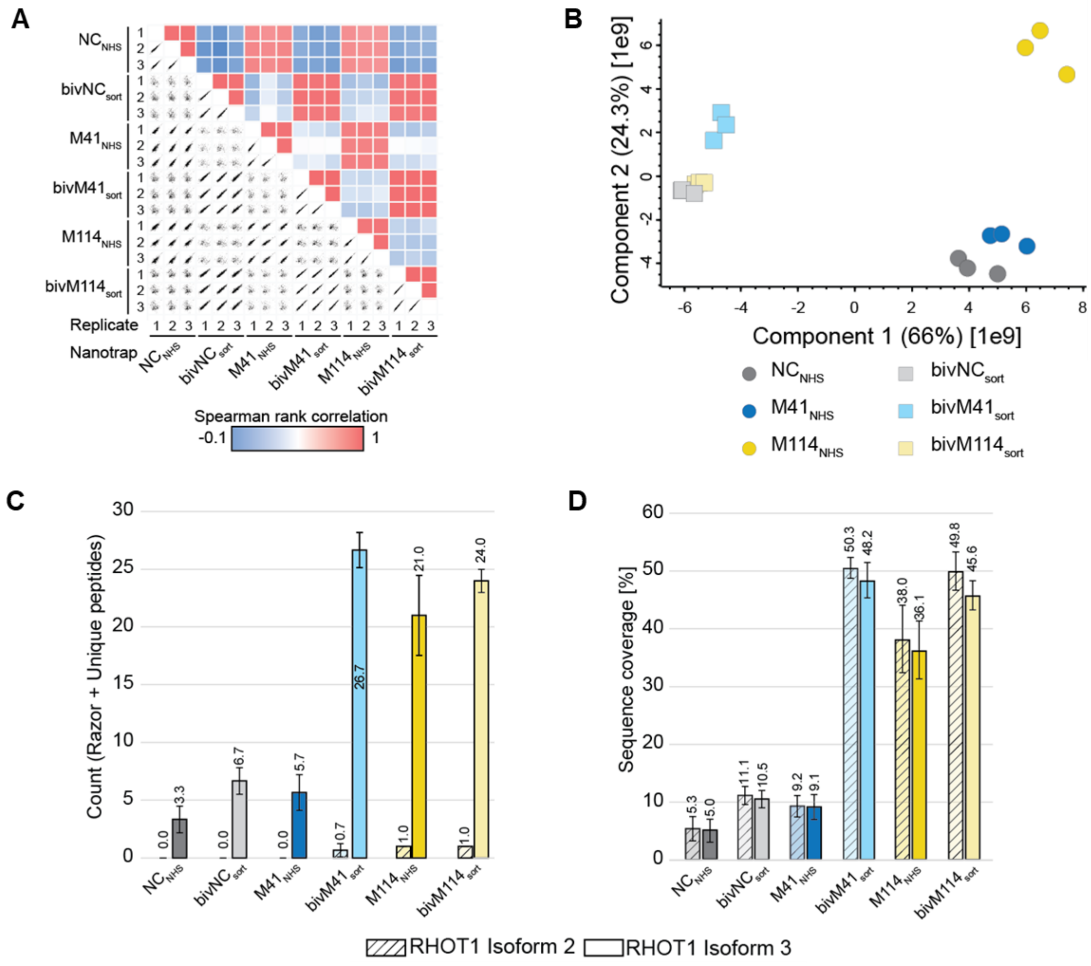

Supplementary Figure 8. Enrichment efficiency of bivalent nanotraps.

(**A**) Multi correlation between replicates and nanotraps. High Spearman rank correlation between replicates and mono- or bivalent nanotraps. (**B**) Principle component analysis (PCA) reflects highest similarity between replicates. 66% of variance between samples explained by nanotrap valency. (**C**) Identification of Miro1 (gene name: RHOT1) specific peptides (razor and unique) for both Miro1 isoforms. (**D**) Averaged sequence coverage of Miro1 (gene name: RHOT1) after precipitation with indicated nanotraps. (**C, D**) Shown are the results from three technical replicates (n = 3) ± S.D.

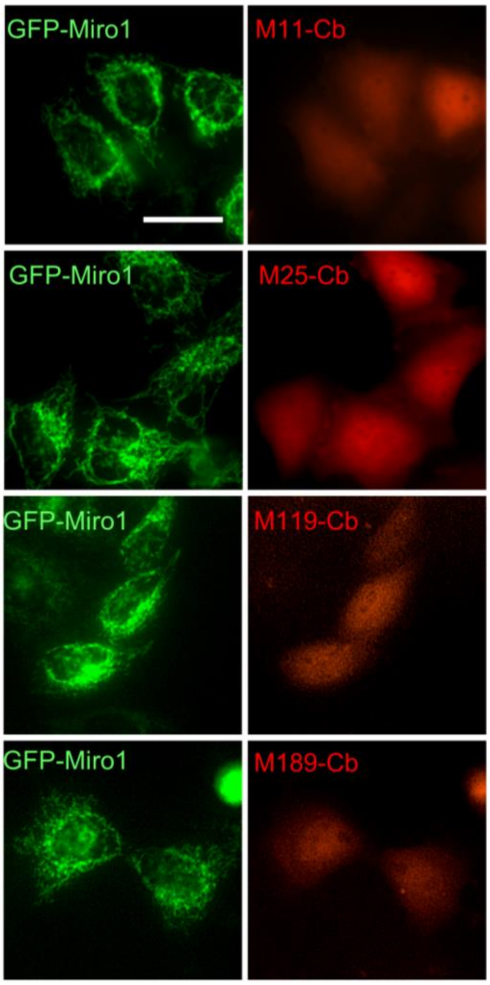

Supplementary Figure 9. Detection of intracellular binding capacities of Miro1-Cbs. Representative fluorescence images of live HeLa cells transiently expressing GFP-Miro1 (left column) in combination with TagRFP-labelled Miro1-Cbs (right column). Scale bar 20 µm.

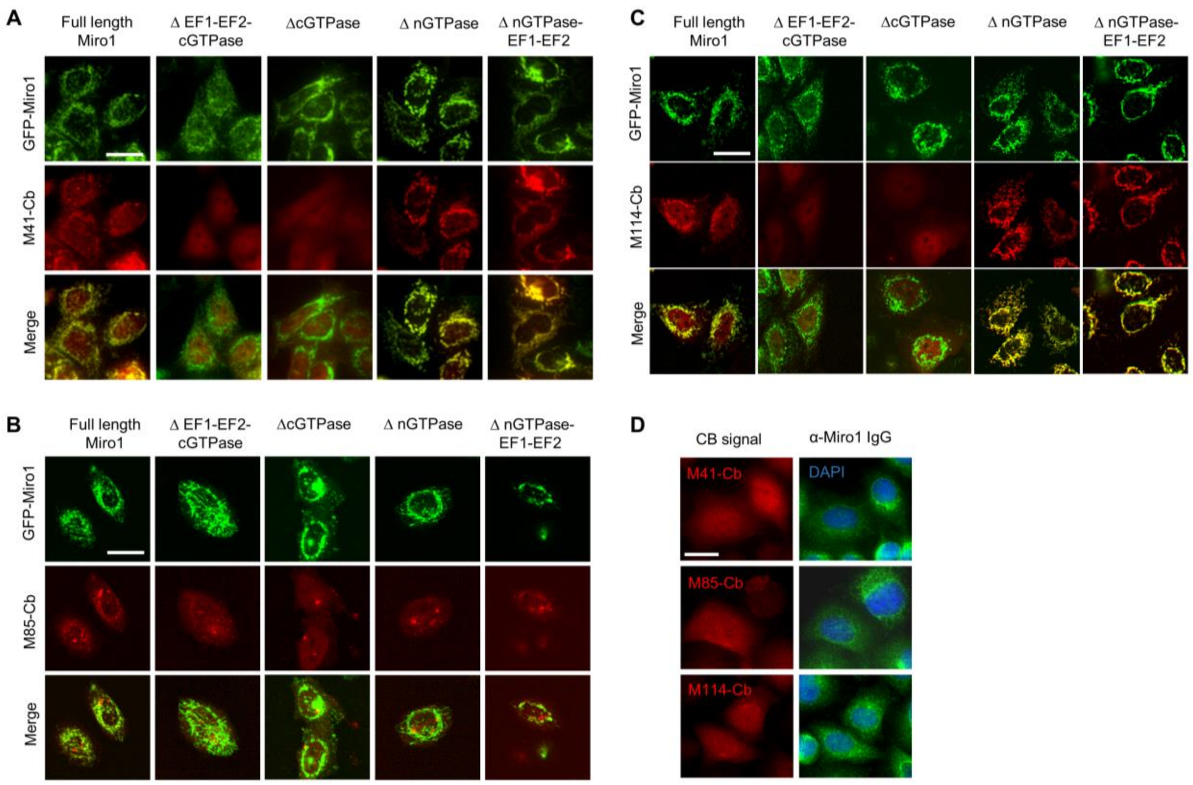

Supplementary Figure 10. Intracellular characterization of domain specific binding of selected Miro1-Cbs.

Representative fluorescent images of live HeLa cells transiently expressing GFP-Miro1 and indicated GFP-tagged Miro1 domain deletion constructs (top row) in combination with TagRFP-labelled M41-Cb (**A**), M85-Cb (**B**) or M114-Cb (**C**) (middle row). Scale bar 20 µm. (**D**) Immunofluorescence detection of endogenous Miro1 in HeLa cells expressing M41-, M85- and M114-Cb (left panel) using an anti-Miro1 antibody (right panel). Scale bar 25 µm.

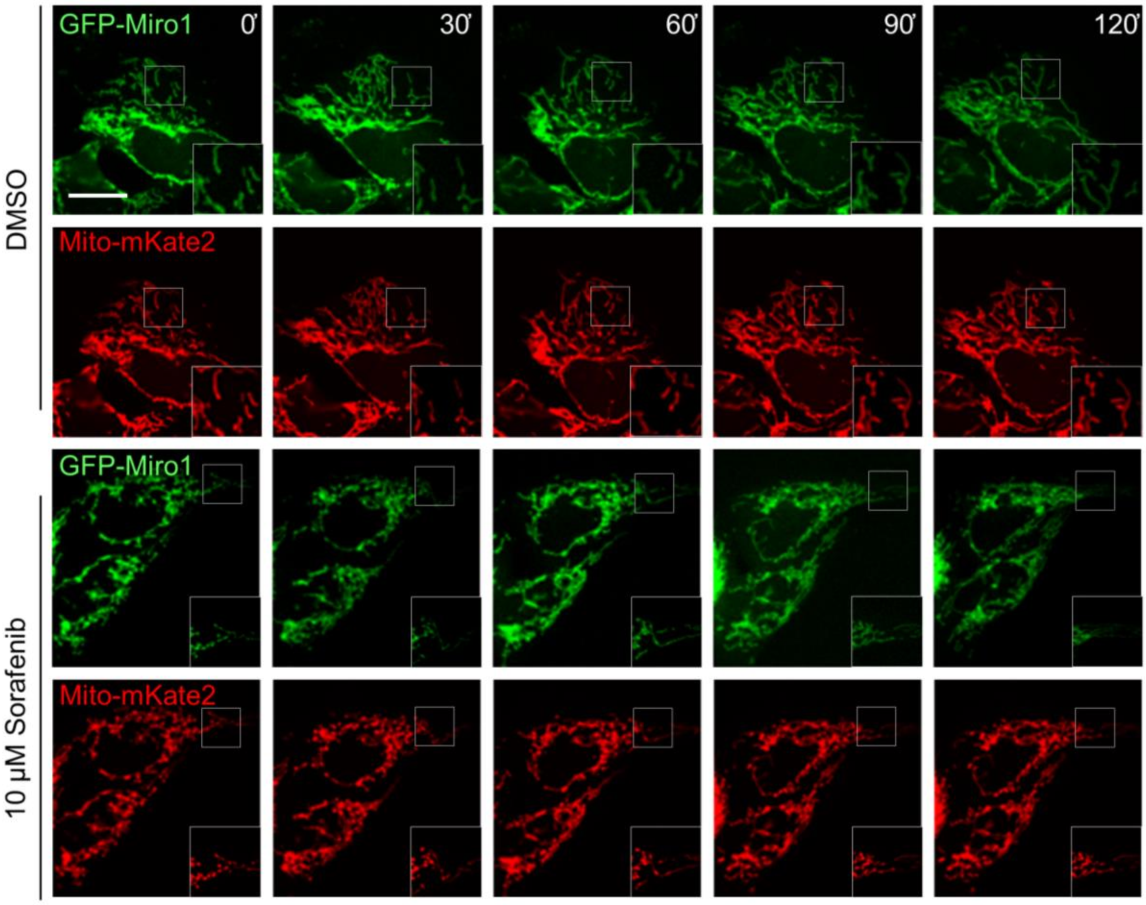

Supplementary Figure 11. Live-cell imaging of mitochondrial phenotypes.

Time-lapse microscopy of U2OS cells transiently expressing GFP-Miro1 and mitoMkate2 (as a mitochondrial marker). To visually track morphological mitochondrial changes, cells were treated with either DMSO as a control (top two rows) or 10 µM Sorafenib (bottom two rows) followed by time-lapse imaging over a 2 hour period. Shown are representative images of three biological replicates. Scale bar 25 µm. Squares at the bottom right represent enlargements of the selected image section.

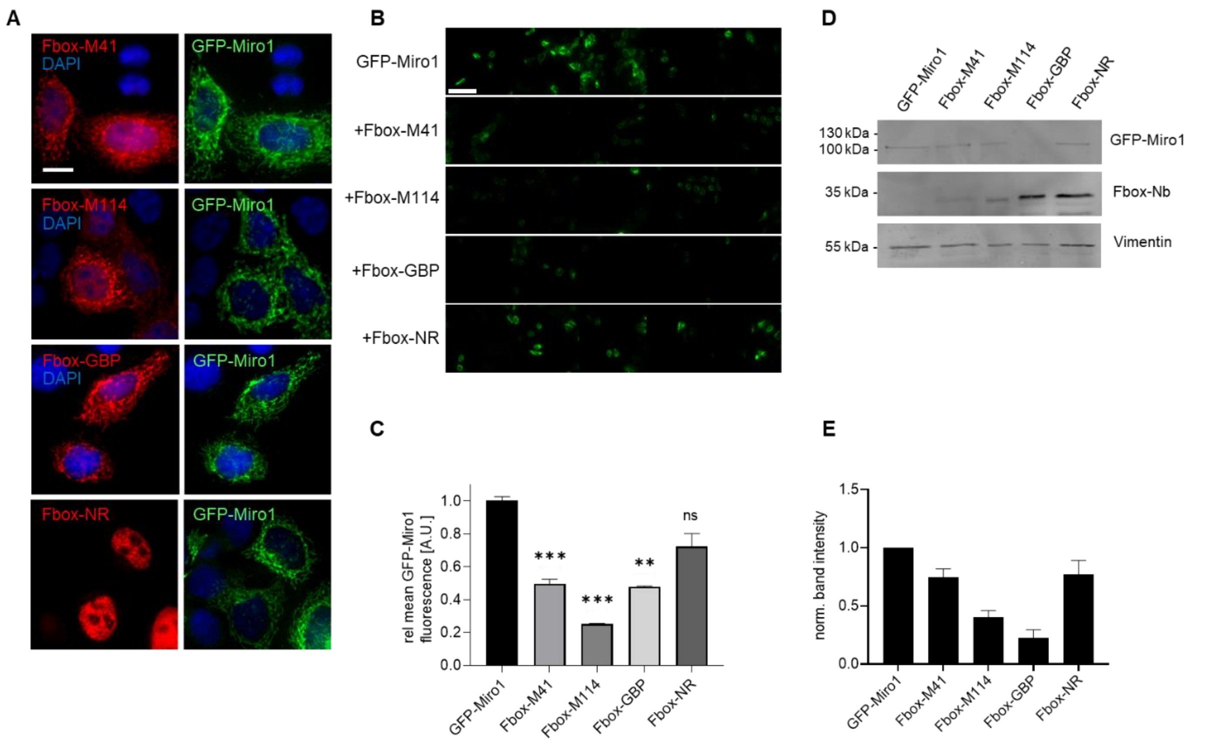

Supplementary Figure 12. Targeted degradation of GFP-Miro1 by Fbox-Nb-based degrons in live cells.

(**A**) Representative confocal images of HeLa cells transiently co-expressing GFP-Miro1 and indicated Miro1-specific Fbox-Nbs (Fbox-M41, Fbox-M114) or a non-related Fbox-Nb (Fbox-NR) construct are shown. For quantitative IF analysis, cells were fixed and permeabilized 24 h after transfection followed by staining with Cy5 conjugated anti-VHH antibody and DAPI. Fbox-Nb expressing cells were subjected to automated image analysis and quantification as described in Material and Methods. Scale bar 20 µm. (**B**) Representative fluorescence images of HeLa cells coexpressing GFP-Miro1 and Fbox-Nb constructs. Scale bar 100 µm. (**C**) Mean GFP-Miro1 fluorescence intensity from HeLa cells co-expressing GFP-Miro1 and Fbox-Nbs constructs were determined by quantitative fluorescence imaging. Fluorescence intensity values were calculated from three samples (n= 3; >500 cells) and normalized to the GFP-Miro1 signal intensity (set to 1). Positive control; GFP-specific Fbox-Nb (Fbox-GBP), negative control; non-related Fbox-Nb construct (Fbox-NR). Data are represented as mean ± SEM. For statistical analysis, student‘s t-test was performed, $^{**}p < 0.01$, $^{***}p < 0.001$. (**D**) Representative immunoblot of lysates of HeLa cells expressing either GFP-Miro1 alone or in combination with

the indicated Fbox-Nb constructs. For analysis 10 µg of the soluble fractions were subjected to SDS-PAGE and immunoblotting followed by detection using an anti-GFP antibody (GFP-Miro1) and an anti-VHH antibody (Fbox-Nb constructs). As loading control, endogenous vimentin was detected using an anti-Vimentin antibody. (**E**) Densitometric evaluation of immunoblot analysis. GFP-Miro1 signals from the different cell lysates were normalized to the GFP-Miro1 signal derived from cells expressing GFP-Miro1 only. Shown are the mean signals from three independent experiments ± S.D.

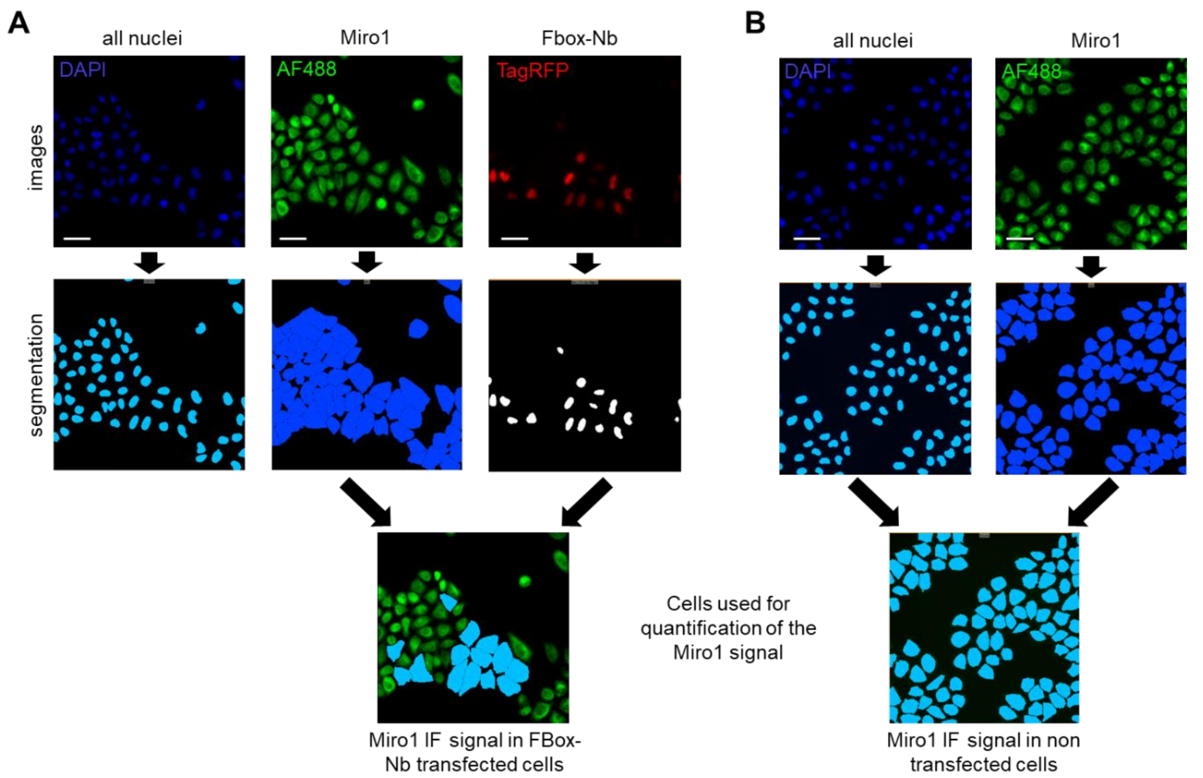

Supplementary Figure 13. Workflow showing automated image segmentation used for quantitative immunofluorescence analysis

Representative images of Fbox-Nb transfected (**A**) or untransfected HeLa cell (**B**) fixed and stained with DAPI and an anti-Miro1 antibody (top row). Image segmentation of stained areas showing the Miro1 signal and the nuclear TagRFP signal indicating degron (Fbox-Nb) transfected cells (middle row). Selection of FBox-Nb expressing cells used for quantification of Miro1 specific antibody signal (bottom row). Scale bar = 50 µm

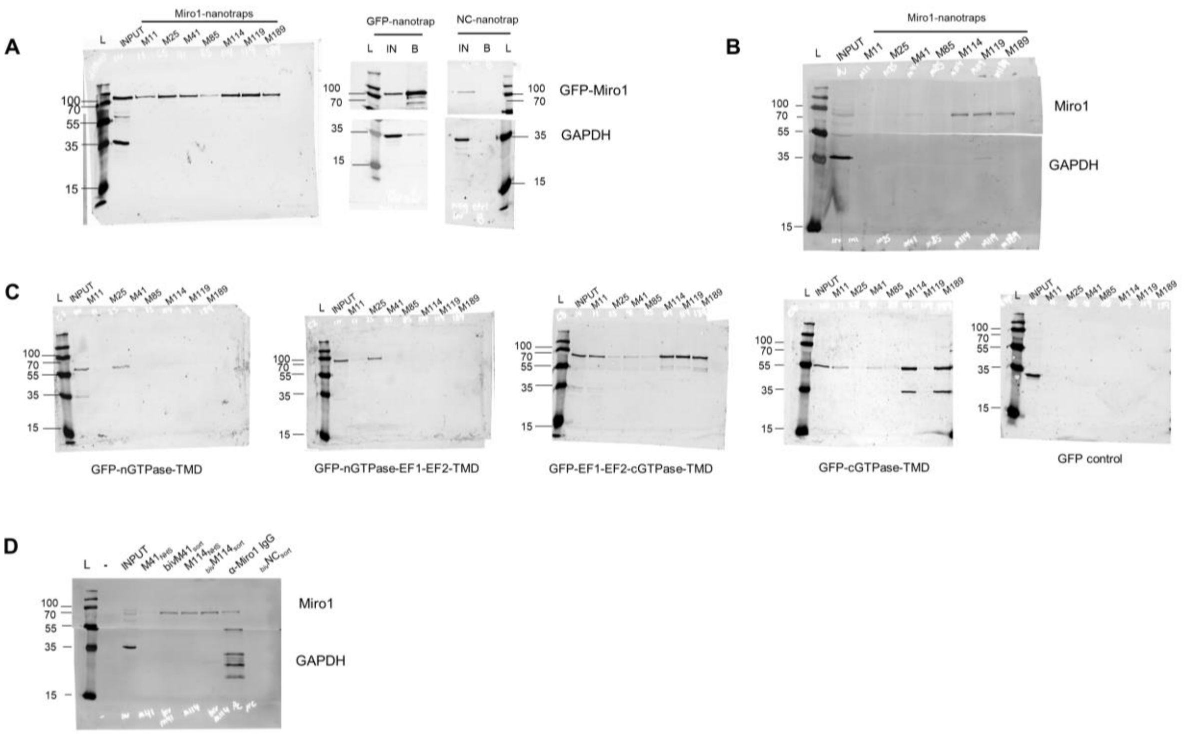

Supplementary Figure 14. Western blot data from immunoprecipitation

(**A**) Full size of Western blots shown in **Figure 2A**, top halves stained with anti-GFP antibody, bottom halves stained with anti-GAPDH antibody (**B**) Full size of Western blots shown in **Figure 2B**, top half detected by anti-Miro1 antibody, bottom half by anti-GAPDH antibody. (**C**) Full size of Western blots shown in **Figure 3B**, detection with anti-GFP antibody. (**D**) Full size of Western blots shown in **Figure 5**, top half detected by anti-Miro1 antibody and the bottom half by anti-GAPDH antibody.

Supplementary Table S1. List of oligonucleotides used in this study

Name	Sequence 5´- 3´	purpose
CALL001	GTCCTGGCTGCTCTTCTACAAGG	Nb library generation
CALL002	GGTACGTGCTGTTGAACTGTTCC	
FR1-1	CATGGCNSANGTGCAGCTGGTGGANTCNGGNGG	
FR1-2	CATGGCNSANGTGCAGCTGCAGGANTCNGGNGG	
FR1-3	CATGGCNSANGTGCAGCTGGTGGANAGYGGNGG	
FR1-4	CATGGCNSANGTGCAGCTGCAGGANAGYGGNGG	
FR1-ext1	GTAGGCCCAGCCGGCCATGGCNSANGTGCAGCTGGTGG	
FR1-ext2	GTAGGCCCAGCCGGCCATGGCNSANGTGCAGCTGCAGGA	
FR4-1	GATGCGGCCGCNGANGANACGGTGACCNGNRYNCC	
FR4-2	GATGCGGCCGCNGANGANACGGTGACCNGNGANCC	
FR4-3	GATGCGGCCGCNGANGANACGGTGACCNGRCTNCC	
FR4-4	GATGCGGCCGCRCTNGANACGGTGACCNGNRYNCC	
FR4-5	GATGCGGCCGCRCTNGANACGGTGACCNGNGANCC	
FR4-6	GATGCGGCCGCRCTNGANACGGTGACCNGRCTNCC	
Miro1fragB_for	CAACAATGGCCATGTATCCACACGTGACACAAGCTGACCTCA	Cloning of GFP-Miro1 mammalian expression construct
Miro1fragB_rev	CAAGCTCTTCAGCAATATCACGG	
Miro1fragA_for	CGTGTGGATACATGGCCATTGTTGTCAATTTAACAAAGA	
Miro1fragA_rev	TGGTGGCGGAGGTAGCATGAAGAAAGACGTGCGGATC	
vectorGA_for	TGGCTACCCGTGATATTGCTG	
vectorGA_rev	CGTCTTTCTTCATGCTACCTCCGCCACCACTTC	
nGTP_for	ATTGGTACCTTTTGGCTTCGAGCAAGTTTTGG	Cloning of Miro1 domain deletion constructs
nGTP_rev	ATTGGTACCCTCCTTCTCCTCTGGGC	
nGTPEF2_rev	GCGGGTACCATTTCTTTGAGTTTGTTTTTTCTGCAGG	
Delta nGTP_for	ATTGGTACCGAGGAGAAGGAGATGAAACCAGC	
Delta nGTP_rev	GCTGGTACCCATGCTACCTCCGCCACC	
cGTP_for	AAAAAACAAACTCAAAGAAATGTGTTCAGATG	
cGTP_rev	CATGCTACCTCCGCCACCACTTCC	
bivM114GA_for	GCCGGCGTGCACTCTGAGGTACAGCTGCAGGAGTCGGG	Cloning of $_{biv}$M114 expression construct
nterm1273_rev	CACCACCGCCAGATCCACCGCCACCTGATCCTCCGCCTCCGCTGCTAACGGTGACC	
bivM114GA2_for	GGTGGATCTGGCGGTGGTGGAAGTGGTGGCGGAGGTAGTGAGGTACAGCTGCAGGAGTCGGG	
downEcoRI_rev	GTTGTAAAACGACGGCCAGTG	
bivM114FPCR_for	GTCTGTGACCGCCGGCGTGCACTCTGAG	
bivM114FPCR_rev	TTTAATTAAGCGGCCGCGAATTGTTGTAAAACGACGGCCAGTG	
NM95_for	TAATCTAGAGGGCCCTATTCTATAGTG	Cloning of Fbox-M41 , Fbox-M114 and Fbox-NR constructs
NM95_rev	GCTGGAGACGGTGACCTG	
frag2IRES_for	CCCAGGTCACCGTCTCCAGCTAACTAGAGGTTAACGAATTC	
frag2IRES_rev	GGTTGTGGCCATATTATC	
nls-insert_for	AAGAAGAGGAAGGTTTGAGCGGCCGCGACTCTA	
nls-insert_rev	CTTAGGGCTGCCTCCATTAAGTTTGTGCCCCAGTTTGCTAG	
frag3-tRFP-nls_for	ATGATAATATGGCCACAACCATGGTGTCTAAGGGCGAAG	
frag3-tRFP-nls_rev	GAATAGGGCCCTCTAGATTAAACCTTCCTCTTCTTCTTAGG	

Supplementary Table S2. Amino acid sequences of all selected Miro1-Nbs.

Miro1-Nbs	Amino acid sequence
M11	QVQLVESGGGSVQPGGSLRLSCAASGFSFSSNVMSWARQAPGKGLEWVSGIYV<br>DGRTHYADSVKGRFTISRDNAKNTVYLQMNSLKPEDTAVYYCAAAGDLSGYSFSP<br>YDYDYWGQGTQVTVSS
M25	HVQLVESGGGLVQPGGSLRLSCIASGFTFSDVGMAWYRQIPGKERDMVASITSFG<br>DQTSYAHSVKGRFTISRDNAKNTVYLQMNTLIPDDTAMYYCTTVLGRERRWGQG<br>TPVTVSS
M41	EVQLVESGGGLVHPGGSLRISCAASGFSFNSSAMSWARQAPGKGLEWVSGIQAD<br>GTTGYAHSVKGRFNISRDNAKNIVYLQMSSLKPEDSAVYYCAKEGWSTVVFGSRD<br>YWGQGTQVTVSS
M85	DVQLVESGGGKVQSGGSLRLSCAASGSKLDDYSIGWFRQAPGKGREGISCITMK<br>TGSFEYVDSVKGRFTISADNAKNTVHLQMNNLKPEDTGIYYCAALRSRGLFCVTSP<br>YEYDLWGQGSPVTVSS
M114	EVQLQESGGGLVQPGGSLRLSCTASGFPVSSAAMSWARQSPGKELEWVSGIYT<br>DGSADYADSLKGRFTISRDDVENTINLQMNSLKPEDTAVYYCKTDWWAGPSGYV<br>AVWGQGTLVTVSS
M119	DVQLVESGGGLVQPGGSLRLSCATSGFPLDNYAIGWFRQAPGSEREGVSCISSS<br>GSYFPGSGSRTHYAHSVKGRFTISRDGAKNTVYLQMDNLKPEDTAVYYCAAVPV<br>PSIRTAETMCVRGSLSEFISWGQGSQVTVSS
M189	EVQLVESGGGLVQPGGSLRLSCTASGFPVSSAAMSWARQSPGKELEWVSGIYTD<br>GSADYADSLKGRFTISRDDVENTINLQMNNLKPEDTAIYYCNANFFEGSWYDYWG<br>QGSPVTVSS

Supplementary Table S3. MS Dataset of endogenous Miro1 enrichment by Miro1 monovalent and bivalent nanotraps (.xlsx file)

(**A**) Description of terminologies in the Miro1 nanotraps MS proteomic analysis Dataset (**B**) Protein Groups enriched by bivM41$_{sort}$ and bivM114$_{sort}$ nanotraps (**C**) Protein Groups enriched by monovalent M41$_{NHS}$ and M114$_{NHS}$ nanotraps. (**D**) Enrichment levels of Ras GTPases by bivM41$_{sort}$ and bivM114$_{sort}$ nanotraps. (**E**) Enrichment levels of Ras GTPases by monovalent M41$_{NHS}$ and M114$_{NHS}$ nanotraps.
